# To Correspondents

**Published:** 1870-09

**Authors:** 


***To Correspondents.***

We cannot undertake to write out opinions on cases presented from practice, for which we are frequently solicited, without the usual *honorarium.* But if such cases are reported in shape for publication in the *Journal*, we will reply through its pages, or ask our correspondents to do so. It has become necessary for us to make this announcement, because the matter of answering letters upon all conceivable (and some inconceivable) cases, has become burdensome to a degree which none but an editor can appreciate.

